# The Single Wire Ureteral Access Sheath, Both Safe and Economical

**DOI:** 10.1155/2016/6267953

**Published:** 2016-11-15

**Authors:** Joan C. Delto, George Wayne, Ajaydeep Sidhu, Rafael Yanes, Akshay Bhandari, Alan M. Nieder

**Affiliations:** Columbia University Division of Urology, Mount Sinai Medical Center, 4302 Alton Road, Suite 540, Miami Beach, FL 33140, USA

## Abstract

*Introduction*. Novel disposable products for ureteroscopy are often inherently more expensive than conventional ones. For example, the Cook Flexor^©^ Parallel™ (Flexor) access sheath is designed for ease and efficiency of gaining upper tract access with a solitary wire. We analyze the cost combinations, efficiency, and safety of disposable products utilized for upper tract access, including the Flexor and standard ureteral access sheath.* Methods*. We performed a retrospective review from January 2014 to October 2014 of patients undergoing URS for nephrolithiasis, who were prestented for various reasons (e.g., infection). Common combinations most utilized at our institution include “Classic,” “Flexor,” and “Standard.” Total costs per technique were calculated. Patient characteristics, operative parameters, and outcomes were compared among the groups.* Results*. The most commonly used technique involved a standard ureteral sheath and was the most expensive ($294). The second most utilized and least expensive combination involved the Flexor, saving up to $80 per case (27%). All access sheaths were placed successfully and without complications. There were no significant differences in operative time, blood loss, or complications.* Conclusions*. In prestented patients within this study, the Flexor combination was the most economical. Although the savings appear modest, long-term impact on costs can be substantial.

## 1. Introduction

Ureteroscopy for the treatment of nephrolithiasis is both a mainstay of care and a driver of its costs. A wide variety of commercially available disposable products for ureteroscopy can be utilized in numerous combinations to reach similar surgical objectives with high stone-free rates and minimal complications and secondary procedures. The abundance of tools and technologies provides surgeons the creativity to perform successful and efficient ureteroscopic surgeries [1, 2]; however, technological advancements, such as decreasing scope diameter and innovative disposables, drive costs ever higher [3]. The selection of such expensive materials, their unnecessary opening by operating room staff, and the wealth of disposable supplies are oft-cited culprits in ureteroscopy costs.

Numerous institutions have increased their efforts to reduce spending [4–6]. Overall, they attempted to improve ureteroscopy maintenance and repair costs, minimize costs of disposables, and utilize economical operative techniques [4–6]. Variable costs for nonreusable, disposable products (e.g., guidewires and access sheaths) can be quite large. Certain guidewire preference and need for special wires in the event of difficulty bypassing an impacted stone are quite costly. Ureteral access sheaths are also expensive but provide the benefit of continuous upper tract drainage, improved visibility, and easy access to the upper tract with minimal trauma from repeated instrumentation (i.e., basket retrieval of stones). The Cook Flexor Parallel access sheath (Flexor) is a recent addition and does simplify ureteroscopy. Our goal was to evaluate the potential cost impact associated with use of such equipment. We thus reviewed our ureteroscopy database to determine if savings could be found by adjusting one's preferences for such disposable items.

## 2. Patients and Methods

**Figure 1:**
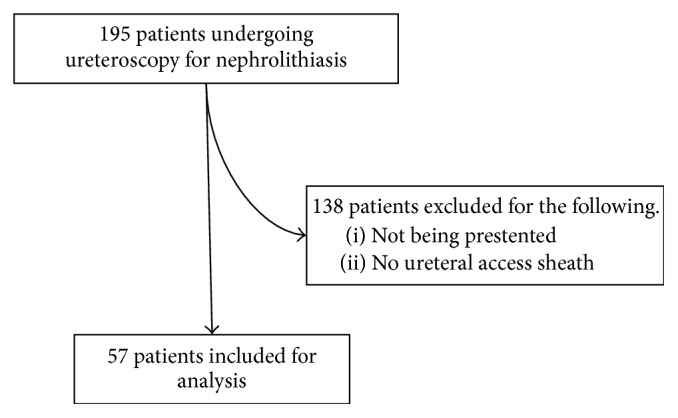
Patient selection, inclusion, and exclusion.

After receiving IRB approval from our institution, we performed a retrospective chart review, identifying 195 ureteroscopy cases for nephrolithiasis performed by multiple surgeons at our institution between January 1, 2014, and October 31, 2014. We attempted to balance our patient selection by specifically evaluating patients with an initial diagnosis of proximal ureterolithiasis or nephrolithiasis who were “prestented” prior to elective ureteroscopy. This cohort of patients was not electively prestented but had prior stenting for emergent drainage of infected stone, severe stone impaction, or a tight ureter not amenable for safe passage of a ureteroscope. Sheaths were often utilized for large proximal stones or stones within the kidney, when the surgeon anticipated longer operative time to fragment and remove the stone (to allow for adequate renal drainage and improved visibility). We identified 57 patients who met these inclusion criteria. We purposefully did not include those patients who had undergone primary ureteroscopy since access sheaths, a target of our analysis, are not often used at the time of such a procedure in our institution. Patients were thus excluded if a ureteral access sheath was not utilized or if they were not prestented (Figure 1).

We obtained patient demographic information, operative parameters (stone characteristics, estimated blood loss, operative time, and intraoperative complications), and perioperative metrics (estimated blood loss, complications, admission, residual stone, and auxiliary procedures). We specifically identified which type and quantity of the following disposables were used per case to obtain upper tract access: a wire, an open-ended ureteral catheter, and an access sheath. A retrograde pyelogram was performed with either an open-ended ureteral or dual lumen catheter. A ureteral access sheath with a safety wire was employed in each case. Wire selection consisted of either sensor or polytetrafluoroethylene (PTFE) wires.

Ureteroscopy disposable inventory was evaluated at our institution and list prices were obtained from the purchasing department. Actual and theoretical techniques of gaining upper tract access using various materials were determined, and total cost per technique was calculated and compared (Table 1). The study population was used to identify the three most commonly used instrument combinations and to compare them by cost and outcome, stone-free rates, operative time, and various metrics of complications.

## 3. Results

We identified 57 procedures, which met our inclusion criteria during the study period. In the study population, the three most common disposable instrumentation options included the “Classic,” “Flexor,” and “Standard Access” combinations (Table 1). The “Classic” method requires a standard 5 F open-ended ureteral catheter, two sensor wires, a dual lumen catheter, and an ACMI standard ureteral access sheath (12–14 F, variable lengths). The “Flexor” combination involves an open-ended ureteral catheter, one sensor wire, and a Flexor access sheath (12–14 F, variable lengths). The “Standard Access” consists of a ureteral catheter, two sensor wires, and a conventional ureteral access sheath. Selection of combination was based solely on surgeon preference.

The Classic method was both the most utilized (*n* = 29, 51%) and the most expensive set of instruments, costing approximately $294. The second most utilized method (*n* = 15, 26%), Flexor, cost approximately $214, making it 27% less expensive than Classic. The Flexor only requires one wire, serving as both a guidewire and safety wire. Furthermore, those few cases in which we replaced a sensor wire with a heavy-duty wire for the Flexor combination saw further cost savings of $166 (44%) compared to the Classic. Of note, the preference at our institution was to use the sensor wire. Our third most commonly used set of instruments, “Standard Access,” cost $248 (*n* = 13, 33%). Elimination of a dual lumen catheter in this group decreased costs by 16% ($47) relative to the “Classic” system. The “Flexor” was still 14% ($34) cheaper than the “Standard” grouping.

Table 2 summarizes baseline characteristics for patients falling into each of the three instrumentation groups; groups appeared comparable. In total, 64 stones were identified: 30 using Classic disposables, 16 with Flexor, and 18 with Standard Access. Stone size, location, and composition did not vary significantly between groups.

Operative parameters did not show any significant difference between the groups. There was no appreciable blood loss for any set of instruments. Complication rate, including postoperative admission, fever, or observation, was negligibly variable across instrumentation groups. No intraoperative complications were observed.

Differences in operative time were analyzed and found to be uncompromised despite varying costs of instrumentation. Ureteroscopy performed with Classic instrumentation took a mean time of 81 minutes, compared to 60 minutes for the Flexor set and 71 minutes for Standard Access (*p* = 0.039); a difference in time was also reflected anecdotally in that the Flexor sheath required the passage of only one wire. Moreover, none of our cases experienced failed Flexor access, such that single wire access never necessitated reverting to two wires. No association was identified between operative time and stone characteristics or between time and the surgeon performing the procedure.

Similarly, stone free rate was not associated with stone location, composition, gender, or surgeon. Although only 40% of patients completed postoperative imaging, stone-free rate—defined as all cases with fragments <3 mm in diameter or no stones on postoperative imaging at four weeks—did not differ significantly between groups. Moreover, no patients in the cohort re-presented with clinical symptoms of stone recurrence.

Regarding immediate postoperative admissions, there were more patients admitted in the Flexor group (*n* = 4) with a mean length of stay of less than one day. Admissions were for observation in the setting of a previous history of urosepsis, pain control, and, lastly, lethargy attributed to anesthesia. Classic incurred three admissions, one each for fever, nausea, and preemptive observation given a history of urosepsis. There was one admission in the Standard Access group for fever. These differences in length of stay were not statistically significant.

## 4. Discussion

We found that using the Flexor sheath and sensor wire combination can incur cost savings of up to $80 per case. Although these savings appear negligible on a case-by-case basis, we estimate a more lucrative cost reduction on a larger scale. For example, studying only the state of California, ureteroscopy for nephrolithiasis had been performed at least 18,933 times in 2010, and cases had generally been increasing since 2005 [7]. We considered ureteroscopic procedures performed only in our single institution and found that between $34 and $80 could be saved per case by simply using a new technology and one less wire. We found that 29% of patients in our study were prestented and later required ureteral access sheaths. Assuming that Californians require ureteral access sheaths as often as our population (29%) and pay similar fees for supplies, we extrapolate that the “Flexor” combination of disposables could have saved them between $187,000 and $439,000 in 2010 alone, with savings increasing in years thereafter [7].

The Cook Flexor Parallel sheath is a relatively new disposable product. Recent studies demonstrated that the Flexor sheath is an effective and safe tool, which requires more force during placement but results in less shearing compared to other single wire systems [8, 9]. Per retail pricing, the Flexor Parallel sheath—although relatively expensive—can result in an overall 27% reduction of disposables costs, as compared to our “Classic” access sheath instrumentation (Table 1).

While we recognize that ureteral access sheaths may be avoided for some cases of ureteroscopy for even further cost savings, they provide the benefit of continuous renal drainage, decreased intrarenal pressure, improved visibility, and easy repeated access to the kidney. We thus utilize access sheaths as frequently as case selection permits at our institution.

We noticed that all of our surgeons utilized the sensor wire as their default wire of choice. Although this wire is more expensive than a standard PTFE wire, the added benefit of this wire is the hybrid hydrophilic nitinol tip with the stiffness of a PTFE coated wire. Anecdotally, surgeons report the advantage of a softer, less traumatizing, gentle wire tip and the durability of the wire that is not prone to the detrimental bending that is experienced with the standard PTFE wire. Moreover, the PTFE wire may not be a suitable safety wire given a high possibility of kinking due to the angle required to exit the Flexor Parallel sheath.

A retrograde pyelogram was done in every case to map renal anatomy and stone location and to ensure proper wire placement. For extra cost reductions, retrograde pyelography may be forgone in placing the guidewire into the orifice, thus eliminating the use of either an open-ended catheter or dual lumen.

In our study, we specifically only included patients who had been prestented (e.g., for infection, acute renal failure) prior to elective ureteroscopy for proximal ureteral or renal stones to be able to normalize our treatment groups. Although patients in our cohort were prestented for infected stone or tight ureters, Chu et al. described the benefits of elective prestenting [10, 11]. Prestenting prior to laser lithotripsy in patients with a stone size greater than 1 cm resulted in a median cost reduction of $10,000 secondary to decreased operative room time and lower rates of reoperation. Moreover, prestenting has been associated with increased stone clearance on postoperative imaging, although statistical significance was not achieved [5, 12].

Although there are many variables influencing stone clearance and operative time including stone characteristics, patient anatomy, and surgeon skill, its efficiency may be attributed to the ease of use and the elimination of multiple steps required for sheath and safety wire placement. Our study demonstrates that the Flexor Parallel sheath is not associated with increased operative time, stone-free rate, complications, or blood loss. Moreover, our results suggest that the flexor parallel sheath may actually be associated with decreased operative time compared to the Classic access, which requires multiple steps to perform a retrograde pyelogram and to place two wires into the renal pelvis. At the least, our analysis suggests that the Flexor combination allows for significant savings in money and several steps (time) during a routine procedure with minimal effort and minimal effect on patient outcomes, anesthesia time, stone-free rate, or rate of complications.

In addition to the savings achieved by using Flexor instrumentation, other combinations of disposables may achieve even lower costs. Theoretically, the most cost-effective method utilizing the items available at our institution would include a ureteral catheter, two heavy-duty wires, and a standard access sheath. Comparative to the Classic combination, this would reduce costs by 48% (38% reduction compared to Standard access); however, this method was not routinely used at our institution and should be evaluated prospectively along with other cost-effective combinations. Importantly, most of the products that are typically available to us, and to other, similarly-sized institutions, are determined by contracts with the purchasing department.

Careful consideration of other disposable materials can result in significant price savings. When utilizing a 5 French open-ended ureteral catheter, the need for a dual-lumen catheter or an additional safety wire is eliminated. A dual lumen catheter provides the surgeon a second channel for retrograde pyelogram and placement of safety wires. However, the convenience of a dual lumen increases costs approximately threefold compared to the use of an open-ended ureteral catheter, which performs the same task in multiple steps. Similarly, regardless of preferred instrumentation, modest behavioral interventions may curtail wasteful spending; at our institution, we mandate that no disposables be opened until requested by the surgeon, eliminating superfluous spending.

Our study does have limitations. We report a single center retrospective study with a moderate number of patients and a relatively short follow-up. Since our study evaluated costs, we did not believe long-term follow-up was necessary. Furthermore, there may be some surgeon variability, as one surgeon predominantly employed the Flexor parallel sheath, and the remaining surgeons utilized the standard ACMI ureteral access sheath; nonetheless, outcomes did not vary by surgeon with statistical significance. Moreover, not all surgeons routinely obtained postoperative imaging, but even then, often patients were noncompliant. We also did not include other disposable products including laser fiber, stone retrieval baskets, or a comparison of surgeons performing the operations, all of which may contribute to stone clearance success rates.

## 5. Conclusion

Ureteroscopy expenses are driven by continually improving technologies, a variety of disposable instruments, and wasteful utilization. Of these, the surgeon can easily control which disposables are utilized. We demonstrated that careful selection of these disposable products for upper tract access could dramatically decrease costs without compromising patient safety or procedural success. In our study, the Flexor combination was the most cost effective, saving between $34 and $80 (14%–27%, relative to Classic and Standard combinations)

Many studies have suggested fairly radical changes to ureteroscopy—not without merit—to save costs. Although future trials would be valuable in further delineating the specifics of our results, we conclude that, without much effort or drastic alteration in our ureteroscopy technique, up to $80 per case ($439,000 in a state the size of California as previously extrapolated and millions nationally) can be saved by simply rethinking one's preference card without fear of harming or affecting the comfort of our patients. Ultimately, surgeons must assess their own instrumentation of disposables to find the ideal most cost effective products.

## Figures and Tables

**Table 1:** Most commonly used techniques for gaining upper tract access.

	Classic	Flexor	Standard access
	*n* = 21	*n* = 11	*n* = 8
Ureteral catheter	×1	×1	×1
Sensor wire	×2	×1	×2
Dual lumen	×1	—	—
Access sheath	×1	—	×1
Flexor parallel sheath	—	×1	—

*Total cost*	$293.86	$213.92	$247.92

**Table 2:** Baseline characteristics for three instrumentation groups.

Number of procedures	Classic	Flexor	Std. access	*p *value	
	*n* = 29	*n* = 15	*n* = 13		
Age (mean)	60	64	58	0.566^*∗*^	
Male	13	9	8	0.485^*∗∗*^	
Procedure type					
Initial surgery	19	10	12	0.176^*∗∗*^	
Second look	10	5	1		
Largest stone (mean, cm)	1	1	1	0.771^*∗*^	
Number of stones (mean)	1	1	2	0.220^*∗*^	
Location of stones					
Ureter	7	6	5	0.468^*∗∗*^	
Pelvis	11	2	3	0.205^*∗∗*^	
Lower pole	8	1	5	0.129^*∗∗*^	
Interpolar	1	3	2	0.192^*∗∗*^	
Upper pole	3	4	3	0.337^*∗∗*^	
Total stones identified	30	16	18		
Laterality					
Left-sided stones	17	10	10	0.510^*∗∗*^	
Right-sided stones	8	4	5	0.739^*∗∗*^	
Bilateral stones	4	2	3	0.714^*∗∗*^	

^*∗*^ANOVA.

^*∗∗*^Pearson chi-squared.
